# Multimodal Prehabilitation for Hernia Repair: Linking Metabolic Modulation and Mechanical Methods

**DOI:** 10.3390/biomedicines13123117

**Published:** 2025-12-18

**Authors:** Dan Nicolae Paduraru, Alexandru Cosmin Palcau, Daniel Ion, Razvan Seicaru

**Affiliations:** 1 Department of General Surgery, Carol Davila University of Medicine and Pharmacy, 050474 Bucharest, Romania; 2 Department of General Surgery, CF2 Clinical Hospital, 011464 Bucharest, Romania; 3 IIIrd Clinic of General and Emergency Surgery, University Emergency Hospital, 050098 Bucharest, Romania

**Keywords:** GLP-1 receptor agonists, hernia repair, preoperative optimization, biomechanically calculated repair, botulinum toxin, progressive pneumoperitoneum

## Abstract

**Background**: Abdominal wall hernias represent a significant global surgical burden, with over 20 million repairs performed annually. The convergence of rising obesity and diabetes rates with complex hernia management has necessitated innovative preoperative optimization strategies that address both metabolic dysfunction and mechanical challenges. **Objectives**: This comprehensive review synthesizes current evidence on emerging pharmacologic and procedural optimization strategies for patients undergoing abdominal wall hernia repair, with particular emphasis on glucagon-like peptide-1 (GLP-1) receptor agonists, botulinum toxin A (BTA) injections, progressive preoperative pneumoperitoneum (PPP) and biomechanical calculated repair. **Methods**: We conducted an extensive literature review incorporating recent clinical trials, observational studies, and meta-analyses, focusing on metabolic optimization with GLP-1 receptor agonists, mechanical preparation techniques, and their comparative effectiveness in reducing perioperative complications and hernia recurrence. **Results**: GLP-1 and GLP-1/GIP agonists demonstrate substantial metabolic benefits including weight reduction (10–20%), improved glycemic control, reduced systemic inflammation, and decreased postoperative complications in surgical populations. Recent evidence suggests reduced surgical site infection, thromboembolic events, and wound dehiscence in GLP-1 receptor agonists users. However, concerns regarding delayed gastric emptying and aspiration risk require careful perioperative management. BTA and PPP remain valuable techniques for mechanical optimization in loss-of-domain hernias, though modern biomechanically calculated repair (BCR) approaches using cyclic load analysis may reduce their necessity in many cases. The GRIP/CRIP concept demonstrates superior outcomes with 5–7% five-year recurrence rates compared to 15% with conventional approaches. Emerging evidence highlights collagen metabolism dysfunction as a fundamental determinant of hernia recurrence, prompting development of collagen-focused prehabilitation programs incorporating nutritional supplementation, aquatic exercise, and targeted physical conditioning. **Conclusions**: A paradigm shift toward integrated, personalized preoperative optimization is emerging, combining metabolic conditioning with mechanical preparation based on individual patient phenotypes and hernia complexity. Future research should focus on comparative effectiveness trials, optimal timing protocols, and multimodal strategies to maximize surgical outcomes while minimizing complications.

## 1. Introduction

### 1.1. Global Epidemiology and Burden

Current epidemiological data indicate that more than 20 million hernia repairs are performed globally each year, with substantial regional variation: North America and Europe report the highest per-capita repair rates (approximately 150–300 per 100,000 population annually), while lower-income regions demonstrate significantly lower rates likely reflecting both reduced surgical access and underreporting [1,2,3]. Inguinal hernias alone account for approximately 75% of all abdominal wall hernias, with lifetime risk estimated at 27% in men and 3% in women [4,5]. The substantial economic burden associated with hernia repair exceeds €3 billion annually in Europe alone, including direct surgical costs, postoperative care, and management of recurrence, underscoring the importance of optimizing surgical outcomes through comprehensive preoperative strategies [5].

Incisional hernias represent a particularly challenging subset, occurring in 10–20% of patients following abdominal surgery, with rates escalating to 30% in high-risk populations [6,7]. Recent data from the French national database revealed that among 431,619 patients who underwent laparotomy, 5% required incisional hernia repair within five years, increasing to 17% after digestive surgery [8]. The five-year recurrence rate following ventral hernia repair exceeds 40% in patients with mesh repair and approaches 70% in those without mesh reinforcement, highlighting the critical need for effective preoperative optimization strategies [9,10].

### 1.2. The Metabolic-Mechanical Convergence

The contemporary management of abdominal wall hernias is increasingly complicated by the global epidemic of metabolic disease. According to the World Health Organization, over 2.5 billion adults are overweight, with more than 890 million classified as obese [11]. Simultaneously, the International Diabetes Federation reports that 589 million adults worldwide live with diabetes, projected to reach 853 million by 2050 [12,13]. Both conditions represent independent risk factors for poor surgical outcomes, including impaired wound healing, surgical site infections, hernia recurrence, and prolonged hospitalization [14,15,16].

The pathophysiological intersection of obesity, diabetes, and hernia formation creates a complex clinical scenario. Excess visceral adiposity increases intra-abdominal pressure (IAP) [17], which directly contributes to both primary hernia development and recurrence following repair [18]. Obesity has been identified as the principal risk factor for incisional hernia development, with repair rates reaching 31% after digestive surgery in obese patients [8,19]. Meta-analyses have confirmed that obesity (BMI > 30 kg/m^2^), smoking, diabetes mellitus, chronic obstructive pulmonary disease (COPD), ASA grade III-IV status, and corticosteroid use all significantly increase the odds of hernia recurrence (OR ranging from 1.34 to 2.08) [20].

### 1.3. The Evolution of Preoperative Optimization

Traditional preoperative optimization strategies have relied predominantly on lifestyle modification, insulin therapy, and restrictive dieting. However, these conventional approaches demonstrate variable effectiveness and face implementation challenges. Systematic reviews meta-analysis for lifestyle modification programs alone, such as low-calorie diets. report mean weight loss of only 3.64 kg (95% CI −2.07–9.35) substantially below the weight reduction required for meaningful surgical risk reduction [21]. This has catalyzed the development and adoption of novel pharmacologic and procedural interventions designed to accelerate and enhance preoperative preparation [22,23].

Contemporary hernia surgery guidelines from the European Hernia Society (EHS) and American Hernia Society (AHS) recommend delaying elective repair in high-risk patients until appropriate optimization is achieved [24]. However, the optimal methods, timing, and patient selection criteria for various optimization strategies remain subjects of ongoing investigation and debate [25,26].

Recent years have witnessed two parallel revolutions in hernia surgery that fundamentally challenge traditional optimization paradigms: biomechanically calculated repair and collagen metabolism as central pathophysiology. The development of GRIP (Gained Resistance to Impacts related to Pressure) and CRIP (Critical Resistance to Impacts related to Pressure) concepts represents a paradigm shift from empiric repair techniques to individualized, biomechanically optimized approaches [27].

By assessing abdominal wall stability under cyclic loading conditions—the 23,000+ daily breathing cycles and countless movements involving increased intra-abdominal pressure—these methods enable quantitative prediction of repair durability [28]. Registry data from the STRONGHOLD group demonstrate five-year recurrence rates below 5% with biomechanically calculated approaches, compared to approximately 15% with conventional techniques in experienced hernia centers—a three-fold improvement representing one of the most significant advances in hernia surgery outcomes in recent decades [29].

Mounting evidence demonstrates that hernia formation and recurrence fundamentally reflect systemic extracellular matrix (ECM) dysfunction rather than purely mechanical failure. Patients with recurrent hernias exhibit profoundly altered collagen I/III ratios and systemic alterations in collagen turnover biomarkers [30]. This recognition has catalyzed development of collagen-focused prehabilitation programs incorporating nutritional supplementation (hydrolyzed collagen peptides, vitamin C, zinc, copper) and targeted physical conditioning aimed at improving tissue quality prior to repair [31,32].

### 1.4. Objectives and Scope

This comprehensive review aims to synthesize current evidence on emerging preoperative optimization strategies for abdominal wall hernia repair, with particular emphasis on:

1. Pharmacologic metabolic optimization: Detailed examination of GLP-1 receptor agonists and dual GLP-1/GIP receptor agonists, including mechanisms of action, clinical efficacy, safety considerations, and perioperative management protocols
2. Mechanical preparation techniques: Analysis of botulinum toxin A injections and progressive pneumoperitoneum for abdominal domain expansion
3. Comparative effectiveness: Critical evaluation of pharmacologic versus procedural approaches across different patient populations and hernia types
4. Integration strategies: Discussion of multimodal optimization protocols combining metabolic and mechanical interventions
5. Future directions: Identification of research gaps and emerging trends in preoperative hernia optimization

## 2. Methods

This comprehensive narrative review synthesized current evidence on preoperative optimization strategies for abdominal wall hernia repair. We conducted systematic literature searches in PubMed, Embase, Scopus, and Cochrane Library databases from inception through January 2025. Search terms included: (“GLP-1” OR “semaglutide” OR “tirzepatide” OR “liraglutide” OR “dulaglutide”) AND (“hernia” OR “abdominal wall” OR “ventral hernia” OR “incisional hernia”) AND (“preoperative” OR “perioperative” OR “optimization”); (“botulinum toxin” OR “BTA”) AND (“hernia” OR “component separation”); (“pneumoperitoneum” OR “PPP”) AND (“hernia” OR “loss of domain”); (“biomechanical” OR “GRIP” OR “CRIP”) AND “hernia repair”; (“collagen” OR “extracellular matrix”) AND (“hernia” OR “prehabilitation”).

We included randomized controlled trials, prospective and retrospective cohort studies, systematic reviews, meta-analyses, case series (≥10 patients), and relevant case reports for emerging safety concerns. Studies were excluded if they: (1) were published in languages other than English, (2) contained insufficient methodological detail for quality assessment, or (3) focused exclusively on pediatric populations.

## 3. Pathophysiological Foundations: The Hernia-Metabolism-Mechanics Triad

### 3.1. Metabolic Dysfunction and Hernia Pathogenesis

#### 3.1.1. Obesity and Intra-Abdominal Pressure

Central adiposity exerts profound mechanical and metabolic effects on the abdominal wall [33]. Elevated IAP, directly proportional to visceral adipose tissue volume, creates chronic tension on fascial structures, impairs fascial healing, and predisposes to both primary hernia formation and postoperative recurrence [34,35]. Studies have demonstrated that each unit increase in BMI correlates with progressively elevated IAP and proportionally increased hernia risk [17,34].

The mechanical burden imposed by obesity extends beyond simple pressure effects. Visceral adipose tissue functions as an active endocrine organ, secreting pro-inflammatory cytokines including interleukin-6 (IL-6), tumor necrosis factor-alpha (TNF-α), and C-reactive protein (CRP) [36]. This chronic low-grade inflammation impairs collagen synthesis, disrupts extracellular matrix remodeling, and compromises wound healing—all critical determinants of successful hernia repair outcomes [37,38].

#### 3.1.2. Hyperglycemia and Tissue Healing Impairment

Type 2 diabetes mellitus profoundly influences surgical outcomes through multiple mechanisms. Chronic hyperglycemia induces advanced glycation end-product (AGE) formation, which disrupts collagen cross-linking and compromises tensile strength of healing tissues [39,40]. Additionally, hyperglycemia impairs neutrophil function [41], reduces chemotaxis [42,43], and diminishes phagocytic capacity, thereby increasing susceptibility to surgical site infections [44].

Perioperative glucose control represents a powerful modifiable risk factor. Studies consistently demonstrate that patients with HbA1c levels exceeding 7% experience significantly higher rates of wound complications, infections, and delayed healing [45,46]. Each 1% increase in HbA1c correlates with proportionally elevated complication rates, emphasizing the critical importance of glycemic optimization [47,48].

Nevertheless, the evidence linking hyperglycemia specifically to hernia recurrence remains inconsistent. While perioperative hyperglycemia clearly increases wound infection risk, several large cohort studies have not demonstrated independent associations between diabetes or HbA1c levels and hernia recurrence [49].

#### 3.1.3. Systemic Inflammation and Surgical Risk

The pro-inflammatory state characteristic of metabolic syndrome extends beyond adipose tissue and pancreatic dysfunction [50]. Elevated circulating inflammatory mediators contribute to endothelial dysfunction, prothrombotic states, and impaired tissue perfusion [51,52]—all of which adversely affect postoperative recovery. This systemic inflammatory milieu provides a mechanistic rationale for interventions that simultaneously address metabolic dysfunction and reduce inflammatory burden.

### 3.2. Mechanical Challenges in Complex Hernias

#### 3.2.1. Loss of Domain and Abdominal Compliance

Loss of domain (LOD) hernias present unique mechanical challenges. While no universal consensus exists, LOD is most commonly defined as chronic herniation of >20% of visceral volume outside the peritoneal cavity, though thresholds ranging from 15% to 50% have been reported depending on institutional protocols and measurement methodologies. This definitional variability complicates cross-study comparisons and highlights the need for standardized LOD assessment criteria. Regardless of the precise threshold, LOD results in contracted abdominal musculature, reduced peritoneal cavity volume, and diminished abdominal wall compliance [53]. Reintegration of herniated contents during repair risks acute compartment syndrome, characterized by elevated IAP (>20 mmHg), respiratory compromise, renal dysfunction, and visceral ischemia [54,55].

The pathophysiology of LOD involves progressive adaptation of the abdominal wall musculature to chronically reduced tension. Lateral oblique muscles undergo shortening and fibrosis, while the peritoneal cavity contracts to accommodate reduced visceral volume. This maladaptive remodeling necessitates mechanical interventions to restore abdominal domain and compliance prior to definitive repair [53].

#### 3.2.2. Fascial Tension and Recurrence Risk

Tension at the fascial closure site represents a critical determinant of hernia recurrence [56]. High-tension repairs demonstrate significantly elevated failure rates compared to tension-free techniques. The biomechanical principle underlying successful hernia repair emphasizes the importance of achieving primary fascial closure without excessive tension, supported by appropriately positioned mesh reinforcement [57].

Recent biomechanical studies have quantified the relationship between fascial tension and recurrence risk. Closures performed under tension demonstrate progressive failure rates over time, with tissue ischemia, impaired healing, and eventual dehiscence [58,59]. This evidence underscores the value of preoperative interventions that reduce fascial tension, whether through weight loss, muscle relaxation, or peritoneal cavity expansion.

## 4. GLP-1 and GLP-1/GIP Receptor Agonists: Pharmacologic Metabolic Optimization

### 4.1. Mechanisms of Action and Pleiotropic Effects

#### 4.1.1. Incretin Physiology and Receptor Pharmacology

Glucagon-like peptide-1 (GLP-1) is an incretin hormone secreted by intestinal L-cells in response to nutrient ingestion [60]. GLP-1 receptor agonists mimic endogenous GLP-1, activating GLP-1 receptors expressed throughout the body, including pancreatic β-cells, hypothalamic appetite centers, gastric smooth muscle, and cardiovascular tissues. The resulting effects encompass glucose-dependent insulin secretion, glucagon suppression, delayed gastric emptying, enhanced satiety, and reduced appetite—collectively producing substantial weight loss and improved glycemic control [61].

Dual GLP-1/GIP receptor agonists, exemplified by tirzepatide, represent the next generation of incretin-based therapies. By simultaneously activating both GLP-1 and glucose-dependent insulinotropic polypeptide (GIP) receptors, these agents produce synergistic effects on insulin secretion, glucose homeostasis, and weight reduction, with clinical trials demonstrating superior efficacy compared to GLP-1 monotherapy [62,63,64].

#### 4.1.2. Weight Reduction and Body Composition Changes

The magnitude of weight loss achieved with GLP-1 and GLP-1/GIP agonists substantially exceeds that of conventional interventions. The STEP trials (Semaglutide Treatment Effect in People with obesity) demonstrated mean weight reductions of 15–17% with semaglutide 2.4 mg weekly over 68 weeks [65,66,67,68,69,70]. The SURMOUNT trials with tirzepatide achieved even more impressive results, with weight reductions approaching 20–22% at maximal doses [71].

Critically, the weight loss induced by these agents predominantly reflects reductions in visceral and subcutaneous adipose tissue, with relative preservation of lean body mass [72], even though the results described in the literature are controversial [73]. However, other studies using more precise imaging methodologies (DEXA, MRI) have reported variable lean mass preservation ranging from 60 to 80% of baseline, with significant heterogeneity depending on concurrent exercise intervention, protein intake, and baseline muscle mass [74,75]. This favorable body composition change directly addresses the mechanical burden of obesity on the abdominal wall. Reductions in visceral adiposity decrease IAP, reduce fascial tension, and facilitate tension-free closure during hernia repair.

#### 4.1.3. Glycemic Control and HbA1c Reduction

Beyond weight loss, GLP-1 receptor agonists (e.g., semaglutide, liraglutide, dulaglutide, tirzepatide) produce robust improvements in glycemic control. Semaglutide and tirzepatide consistently reduce HbA1c by 1.5–2.5%, often achieving target levels below 7% in patients with type 2 diabetes. Importantly, these agents exert glucose-lowering effects through glucose-dependent mechanisms, resulting in minimal hypoglycemia risk—a critical safety consideration in the perioperative setting [76,77].

The glycemic improvements achieved with GLP-1 therapy directly translate to reduced surgical risk. Preoperative optimization of HbA1c to <7% correlates with significantly reduced surgical site infection rates, accelerated wound healing, and improved overall surgical outcomes across diverse surgical populations [78].

#### 4.1.4. Anti-Inflammatory and Cardiovascular Benefits

GLP-1 receptor activation extends beyond metabolic effects to encompass potent anti-inflammatory actions. Clinical studies demonstrate significant reductions in circulating inflammatory markers, including CRP, IL-6, and TNF-α, following GLP-1 therapy initiation [79]. These anti-inflammatory effects improve endothelial function, reduce oxidative stress, and enhance tissue perfusion—all beneficial for perioperative outcomes.

Major cardiovascular outcome trials (LEADER with liraglutide, REWIND with dulaglutide, SUSTAIN-6 with semaglutide) have demonstrated significant reductions in major adverse cardiovascular events (MACE) in high-risk populations. For patients undergoing elective hernia repair who often carry substantial cardiovascular risk burdens, these cardioprotective effects represent an additional benefit of preoperative GLP-1 therapy [80,81,82].

### 4.2. Clinical Evidence in Surgical Populations

#### 4.2.1. Observational Studies and Cohort Analyses

While randomized controlled trials specifically evaluating GLP-1 receptor agonists in hernia surgery populations remain limited, substantial indirect evidence derives from studies in related surgical contexts. A landmark 2025 retrospective cohort study analyzed over 13,000 adults with type 2 diabetes undergoing elective surgery, comparing outcomes between GLP-1 users (semaglutide or tirzepatide) and non-users. GLP-1 therapy was associated with:

- 12% reduction in hospital readmissions
- 29% reduction in wound dehiscence
- 56% reduction in thromboembolic events
- Lower rates of overall postoperative complications [83]

A recent study examining total shoulder arthroplasty outcomes in diabetic patients using semaglutide demonstrated significant reductions in multiple adverse outcomes. On multivariable analysis, semaglutide users exhibited 75% lower odds of surgical site infection (OR 0.25), 68% lower odds of cardiac events (OR 0.32), 64% lower odds of venous thromboembolism (OR 0.36), and 75% lower odds of pneumonia (OR 0.25) compared to non-users [84]. These findings suggest broad benefits of preoperative GLP-1 therapy across surgical disciplines.

#### 4.2.2. Bariatric Surgery Literature

The bariatric surgery literature provides valuable insights into GLP-1 agonist effects on surgical candidacy and outcomes. Rubio-Herrera et al. (2023) [85] conducted a retrospective cohort study of 102 patients awaiting bariatric surgery who received liraglutide or semaglutide during the waiting period. After 52 weeks, patients achieved mean weight loss of approximately 17%, and remarkably, nearly 70% no longer required surgical intervention due to sufficient clinical improvement. This study demonstrates that aggressive metabolic optimization may, in select cases, obviate surgical necessity entirely.

#### 4.2.3. Ongoing Clinical Trials

Several ongoing trials are specifically evaluating GLP-1 receptor agonists in preoperative optimization contexts. A registered trial investigating tirzepatide for bariatric optimization is examining effects on surgical risk factors in high-BMI patients [86]. A pilot trial assessed preoperative semaglutide in patients awaiting radical prostatectomy to reduce BMI and visceral adiposity, demonstrating feasibility and patient adherence to GLP-1-based neoadjuvant strategies [87]. While these trials focus on non-hernia populations, their findings will likely inform hernia-specific protocols.

### 4.3. Perioperative Safety Considerations and Controversies

#### 4.3.1. Delayed Gastric Emptying and Aspiration Risk

The most significant perioperative safety concern surrounding GLP-1 receptor agonists involves delayed gastric emptying—a known pharmacologic effect mediated through vagal afferent mechanisms affecting gastric smooth muscle. Multiple case reports have documented retained gastric contents in patients taking semaglutide despite adherence to standard fasting protocols, raising concerns about pulmonary aspiration risk during anesthesia induction [88,89].

Initial guidance from the American Society of Anesthesiologists (ASA) in June 2023 recommended discontinuing GLP-1 receptor agonists one week prior to elective surgery, based on concerns about aspiration risk [90]. However, this recommendation was controversial due to the long half-life of these agents (approximately 7 days for semaglutide), which would necessitate discontinuation for 5+ weeks to ensure complete washout—potentially compromising glycemic control and cardiovascular protection [91,92,93].

Recent evidence has provided important clarification. A large retrospective study by Chen et al. [94] analyzed approximately 6000 patients with GLP-1 RA prescribed preoperative. The study found no significant difference in postoperative respiratory complication rates between GLP-1 users (3.5%) and non-users (4.0%), suggesting that concerns about aspiration risk may be overstated.

However, studies specifically examining gastric contents via endoscopy have yielded more concerning findings. A 2024 study by Santos et al. evaluated residual gastric content (RGC) in patients undergoing esophagogastroduodenoscopy under anesthesia. Among 1094 patients, increased RGC was observed in 20.33% of semaglutide users versus 3.19% of non-users (*p* < 0.001). Importantly, the study identified critical timing thresholds: preoperative discontinuation >21 days in patients with digestive symptoms, and >14 days in asymptomatic patients, resulting in RGC rates similar to non-users [95].

Emerging registry data suggest that absolute aspiration risk remains low (0.8%) even in GLP-1 users, though high-risk phenotypes require identification [96]. Furthermore, agent-specific pharmacokinetics influence risk profiles. Shorter-acting agents like liraglutide (half-life~13 h) may permit shorter discontinuation periods (3–5 days) compared to semaglutide (half-life ~7 days, optimal discontinuation 14–21 days) or tirzepatide (half-life ~ 5 days, discontinuation 10–14 days) in high-risk patients [92,97,98]. Point-of-care gastric ultrasound has emerged as a valuable risk stratification tool, with a standardized protocol demonstrating high sensitivity and specificity for identifying at-risk patients with retained gastric contents [99].

#### 4.3.2. Updated Perioperative Management Guidelines

In 2025, a multi-society consensus emerged with revised guidance acknowledging the complexity of perioperative GLP-1 management [100,101]. The updated recommendations emphasize:

1. Risk stratification: Most patients can continue GLP-1 receptor agonists perioperatively, particularly those undergoing procedures with advanced airway management
2. High-risk identification: Patients with ongoing gastrointestinal symptoms (nausea, vomiting, dyspepsia, bloating) should be considered higher risk
3. Procedural modifications: For high-risk patients, consider liquid diet 36–48 h preoperatively, prokinetic agents, or gastric ultrasound assessment
4. Individualized decision-making: Shared decision-making weighing aspiration risks against benefits of continued therapy (glycemic control, cardiovascular protection)

For elective hernia repair specifically, a reasonable approach involves:

- Continuation of GLP-1 therapy in asymptomatic patients undergoing procedures with endotracheal intubation
- Consideration of 2–3 week discontinuation in patients with gastrointestinal symptoms or those undergoing procedures under moderate sedation
- Utilization of rapid sequence induction and airway protection measures in higher-risk patients [101,102]

### 4.4. Practical Implementation in Hernia Surgery

#### 4.4.1. Proposed Patient Selection Criteria

The following criteria represent expert consensus recommendations pending validation through prospective trials. Ideal candidates for preoperative GLP-1 optimization include:

- Obese patients (BMI > 35 kg/m^2^) awaiting elective hernia repair
- Diabetic patients with suboptimal glycemic control (HbA1c > 7%)
- Patients with metabolic syndrome and elevated cardiovascular risk
- Those requiring delay of surgery for medical optimization
- Patients who have failed conventional weight loss efforts [102,103]

Contraindications and cautions include:

- Personal or family history of medullary thyroid carcinoma or MEN2 syndrome
- History of pancreatitis (relative contraindication)
- Severe gastroparesis or gastrointestinal motility disorders
- Urgent or emergent surgical indications
- Inability to afford therapy or lack of insurance coverage [104]

#### 4.4.2. Proposed Timing and Duration Protocols

Optimal preoperative duration of GLP-1 therapy remains to be definitively established through prospective trials [101]. The following protocols represent extrapolations from bariatric and general surgical literature and require validation:

- Minimum duration: 3–6 months to achieve substantial weight loss and metabolic improvements
- Ideal duration: 6–12 months for maximal benefit, particularly in patients with BMI > 40 kg/m^2^
- Dose escalation: Follow manufacturer protocols for gradual dose titration to minimize gastrointestinal side effects
- Monitoring: Monthly assessment of weight, HbA1c (in diabetic patients), and tolerance [105,106]

## 5. Mechanical Optimization Strategies: Botulinum Toxin and Progressive Pneumoperitoneum

### 5.1. Botulinum Toxin A (BTA) for Chemical Component Separation

#### 5.1.1. Mechanism and Pharmacology

Botulinum toxin type A induces temporary flaccid paralysis of skeletal muscle by inhibiting acetylcholine release at neuromuscular junctions. When injected into the lateral abdominal wall muscles (transversus abdominis, internal oblique, external oblique), BTA produces bilateral muscle relaxation lasting 2–4 months. This “chemical component separation” increases abdominal wall compliance, elongates shortened muscles, and reduces tension required for midline fascial closure [107].

The technique, pioneered by Zielinski et al. in 2013 [108], represents a minimally invasive alternative to traditional surgical component separation techniques. Ultrasound-guided injection ensures accurate delivery to target muscle planes, typically using 100–200 units of onabotulinumtoxin A per side (total 200–400 units) or equivalent doses of other botulinum toxin formulations [108,109].

#### 5.1.2. Clinical Applications and Indications

BTA is primarily indicated for:

- Complex ventral/incisional hernias with defects > 10 cm
- Loss-of-domain hernias with VIH/VAC ratio > 20%
- Recurrent hernias requiring revision surgery
- Patients in whom surgical component separation poses excessive risk [110,111]

Multiple systematic reviews and meta-analyses have evaluated BTA efficacy. A meta-analysis by Timmer et al. [112] demonstrated that preoperative BTA:

- Increases lateral muscle length by 6.3 cm
- Reduces fascial defect width by 3.5 cm
- Improves primary fascial closure rates
- Reduces need for surgical component separation
- May reduce recurrence rates, though long-term data remain limited

#### 5.1.3. Technical Considerations

Optimal BTA protocols involve:

- Timing: Injection 4–6 weeks preoperatively to allow maximal effect (onset 7–10 days, peak effect 2–4 weeks)
- Dosing: Most commonly 100 units per lateral abdominal wall (total 200 units), though doses up to 500 total units have been reported
- Injection technique: Ultrasound-guided injection into each of three muscle layers bilaterally (external oblique, internal oblique, transversus abdominis)
- Assessment: Pre- and post-injection CT imaging to quantify muscle elongation and defect reduction [113,114]

#### 5.1.4. Advantages and Limitations

Advantages:

- Minimally invasive outpatient procedure
- Avoids surgical trauma to abdominal wall planes
- Preserves vascular supply and innervation
- Can be combined with other optimization strategies
- Well tolerated with minimal complications

Limitations:

- Temporary effect requiring timely surgical scheduling
- No systemic metabolic benefits
- Requires procedural expertise and ultrasound guidance
- Cost considerations (approximately 2000 € per treatment)
- Limited utility in small or easily reducible hernias
- Insufficient data for routine use in primary hernias [114,115,116]

### 5.2. Progressive Preoperative Pneumoperitoneum (PPP)

Progressive pneumoperitoneum was first described by Goñi Moreno in 1940–1947 as a method to expand the peritoneal cavity prior to giant hernia repair. The technique involves sequential insufflation of air into the peritoneal cavity over days to weeks, gradually stretching the abdominal musculature and expanding abdominal domain. Despite its long history, PPP remains underutilized, practiced primarily by specialized hernia centers with specific expertise [117,118].

The physiologic rationale for PPP involves:

- Progressive elongation of contracted lateral abdominal muscles
- Expansion of peritoneal cavity volume to accommodate visceral reintegration
- Gradual adaptation of cardiopulmonary function to elevated IAP
- Reduction in postoperative compartment syndrome risk
- Facilitation of tension-free fascial closure [119]

#### 5.2.1. Technical Protocol and Methodology

Catheter placement: A pigtail catheter or specialized access device is placed percutaneously into the peritoneal cavity under ultrasound or CT guidance, typically in a lateral abdominal quadrant away from the hernia defect.

Insufflation protocol: Multiple protocols exist, but typical approaches involve:

- Initial insufflation: 500–1000 cc, titrated to patient tolerance
- Subsequent insufflations: 500–1500 cc every 1–3 days
- Target volume: Variable, based on patient tolerance and volumetric goals (often 8000–15,000 cc total)
- Duration: Typically, 7–21 days, depending on hernia size and patient adaptation

Gas selection: Filtered room air is most commonly used (least expensive, readily available), though some centers prefer carbon dioxide (more rapidly absorbed, potentially lower infection risk).

Monitoring: Daily assessment of vital signs, abdominal circumference, patient symptoms, and insufflated volumes. Some protocols incorporate CT volumetric assessment pre- and post-PPP.

Setting: Can be performed in inpatient, outpatient, or home settings with appropriate patient selection and support systems [120].

#### 5.2.2. Evidence Base and Outcomes

Multiple systematic review examined combined BTA and PPP in loss-of-domain hernias, finding:

- Mean VAC increase
- Reduction in VIH/VAC ratio by up to 15%
- Successful primary fascial closure
- Reduced postoperative respiratory complications
- Lower rates of reoperation for compartment syndrome [111,121]

A prospective study by Bueno-Lledó et al. (2023) reported outcomes in 180 consecutive patients undergoing PPP, with complications occurring in approximately 20% but most being minor and self-limited [122]. A 2023 study analyzing 50 consecutive patients found successful repair in all cases with only three recurrences during follow-up [119].

#### 5.2.3. Complications and Management

PPP-related complications include:

○ Common:
  - Subcutaneous emphysema (most frequent, typically self-limited)
  - Shoulder/abdominal discomfort during insufflation
  - Mild respiratory symptoms
○ Uncommon:
  - Pneumothorax or pneumomediastinum
  - Catheter migration or dislodgement
  - Port site infection
  - Metabolic acidosis (with large air volumes)
○ Rare:
  - Visceral perforation
  - Significant bleeding
  - Cardiovascular decompensation
  - Respiratory failure requiring intervention

Most complications resolve with temporary cessation of insufflations or catheter removal. Serious complications requiring intervention remain rare in experienced hands [123,124].

#### 5.2.4. Combined BTA and PPP Protocols

The synergistic combination of BTA and PPP has gained increasing adoption for complex loss-of-domain hernias. BTA-induced muscle relaxation enhances the elongation achieved by PPP insufflation, potentially producing additive or synergistic effects on abdominal wall compliance. Typically, BTA is administered first (4–6 weeks preoperatively), followed by PPP initiation after BTA effect is established (2–4 weeks preoperatively) [120].

A 2018 prospective study by Bueno-Lledó et al. evaluated 70 consecutive patients treated with combined BTA and PPP, achieving a 16.6% mean reduction in VIH/VAC ratio and successful repair in all patients [125]. A 2022 study by Tang et al. demonstrated that combined therapy enabled successful laparoscopic repair of complex ventral hernias that would otherwise have required open procedures with extensive component separation [126].

## 6. Biomechanically Calculated Repair and Prehabilitation Programs

### 6.1. The GRIP/CRIP Concept: A Paradigm Shift in Hernia Repair

Recent advances in hernia surgery have introduced biomechanically calculated approaches that fundamentally reconceptualize repair planning. The GRIP (Gained Resistance to Impacts related to Pressure) and CRIP (Critical Resistance to Impacts related to Pressure) concepts, developed by Kallinowski and colleagues, represent a revolutionary framework that assesses abdominal wall stability under cyclic loading conditions—mimicking physiological stress from breathing, coughing, and daily activities.

CRIP (Critical Resistance): Defines the minimum biomechanical strength required for durable hernia repair based on defect size, tissue quality, and individual patient factors. CRIP is calculated preoperatively using CT imaging with Valsalva maneuver to assess tissue distension and strain characteristics.

GRIP (Gained Resistance): Quantifies the actual biomechanical strength achieved through a specific repair technique, considering mesh-to-defect area ratio, material properties (tested under cyclic load), fixation methods, and tissue stability. A repair is biomechanically stable when GRIP exceeds CRIP.

The fundamental insight underlying this approach is that the human abdominal wall experiences continuous cyclic loading—estimated at 23,000 breathing cycles daily, plus numerous movements involving increased intra-abdominal pressure. Traditional hernia repair materials and techniques were rarely tested under such dynamic, repetitive stress conditions, potentially explaining persistent recurrence rates despite technical excellence [27].

The STRONGHOLD registry compared biomechanically calculated repair (BCR) to standard repair (SR) techniques using propensity score matching across large patient cohorts. At one-year follow-up, biomechanically controlled repair (BCR) showed significantly fewer complications compared with standard repair (SR), along with lower recurrence rates and markedly reduced chronic pain (median NAS score 0 in the BCR group), while operative time was comparable between techniques once surgeons became familiar with the required calculations [29].

At the three-year follow-up reported by Nessel et al. (2024), biomechanically controlled repair (BCR) demonstrated sustained excellent outcomes in 198 patients with complex incisional hernias, with less than 1% recurrences, consistently low pain levels in both primary and recurrent hernia repairs [28].

Five-year comparative data show that conventional hernia centers report recurrence rates of approximately 15%, whereas the STRONGHOLD BCR approach achieves rates below 5%; this three-fold reduction represents one of the most substantial improvements in hernia surgery outcomes in recent decades [29].

Biomechanical assessment integrates naturally with preoperative optimization strategies, as CT imaging with a Valsalva maneuver can quantify abdominal wall distension—considered stable when expansion is under 1.5 cm—and patients exceeding this threshold may benefit from targeted interventions such as weight loss using GLP-1/GIP agonists to reduce intra-abdominal pressure and tissue strain, botulinum toxin A injections to enhance muscle compliance, progressive preoperative pneumoperitoneum to increase abdominal domain and improve tissue adaptation, and collagen-strengthening protocols to further support structural integrity.

### 6.2. Collagen Metabolism and Prehabilitation Strategies

Emerging evidence fundamentally reframes hernia disease not merely as a mechanical problem but as a manifestation of systemic extracellular matrix (ECM) dysfunction. Multiple studies demonstrate that patients with hernias—particularly recurrent hernias—exhibit profound alterations in collagen metabolism [127].

Several studies have demonstrated that the collagen type I/III ratio within fascial tissues is markedly altered in patients with hernias compared with healthy controls, reflecting a shift toward a mechanically weaker extracellular matrix. This imbalance is even more pronounced in individuals with recurrent hernias, in whom a further reduction in type I collagen relative to type III has been observed, suggesting a more profound and persistent defect in collagen synthesis and remodeling. Collectively, these findings support the concept that impaired connective-tissue quality represents a systemic and progressive substrate predisposing to both primary and recurrent hernia formation [128].

Serum biomarker studies indicate that hernia patients exhibit systemically altered collagen metabolism rather than isolated local defects, with findings showing decreased type III and V collagen turnover in individuals with inguinal hernias, increased basement membrane (type IV) collagen turnover in both inguinal and incisional hernia patients, and systemic persistence of these abnormalities, suggesting underlying genetic or acquired defects in collagen synthesis [30].

A 2025 comparative study examining oncologic versus non-oncologic patients found that collagen disorganization and decreased type I/III ratios strongly correlated with hernia recurrence. Stereomicroscopic analysis using polarized light revealed that collagen fiber organization, birefringence patterns, and structural integrity all predicted recurrence risk independent of surgical technique [129].

Recognition of collagen’s essential role in abdominal wall integrity has led to targeted prehabilitation strategies designed to enhance extracellular matrix quality before hernia repair. Nutritional interventions—including hydrolyzed collagen peptides, vitamin C, zinc, copper, and amino acids such as arginine and glutamine—support collagen synthesis and cross-linking, with clinical evidence showing that multimodal supplementation can increase early postoperative collagen production [31]. Physical conditioning, particularly supervised core-stabilization programs, provides low-impact mechanical stimulation that promotes favorable collagen remodeling while avoiding excessive intra-abdominal pressure. Emerging molecular therapies further aim to optimize ECM biology by modulating MMP activity, enhancing growth-factor-mediated fibroblast activation, and employing PRP to stimulate local collagen deposition [130,131]. In the future, genetic profiling and serum biomarkers of collagen turnover may enable personalized prehabilitation based on individual connective-tissue characteristics.

## 7. Comparative Analysis: Pharmacologic vs. Mechanical Optimization

GLP-1/GIP agonists and mechanical optimization techniques (BTA, PPP) address fundamentally different aspects of hernia pathophysiology [26,132] (Table 1).

## 8. Emerging Evidence and Recent Advances

### 8.1. GLP-1/GIP Formulations and Delivery Systems

The incretin-based therapy landscape continues to evolve rapidly. Approved oral formulation offers an alternative to injectable therapy, potentially improving patient adherence in preoperative optimization protocols [133,134]. However, efficacy is somewhat reduced compared to subcutaneous administration [135].

Extended-Interval Formulations: Development of monthly and bi-monthly GLP-1 formulations may simplify perioperative management and reduce concerns about delayed gastric emptying through more predictable pharmacokinetics [136].

Triple Agonists (GLP-1/GIP/Glucagon): Retatrutide and other triple agonists in late-stage development demonstrate even greater weight loss (up to 24% in phase 2 trials) and may become future options for aggressive preoperative optimization [137,138].

### 8.2. Refined Perioperative Safety Protocols

Recent research has substantially clarified perioperative GLP-1 management:

○ Gastric Ultrasound Assessment: Point-of-care ultrasound assessment of gastric contents is emerging as a valuable tool for risk stratification. Studies suggest that ultrasound can identify patients with retained gastric contents despite fasting, allowing individualized airway management decisions [99].
○ Preoperative Prokinetic Strategies: Some centers are implementing preoperative metoclopramide or domperidone in GLP-1 users undergoing elective surgery to accelerate gastric emptying and mitigate aspiration risk [139,140].
○ Risk Prediction Models: Development of clinical prediction tools incorporating GI symptoms, duration of therapy, dose, and timing of last administration to stratify aspiration risk and guide perioperative management.

### 8.3. Advanced Imaging and Planning Technologies

3D Volumetric Reconstruction: Advanced CT-based volumetric analysis enables precise quantification of:

- Visceral in hernia (VIH) volume
- Total visceral abdominal content (VAC) volume
- VIH/VAC ratio for LOD assessment
- Pre- and post-optimization changes in abdominal domain
- Personalized prediction of closure feasibility [141,142]

Computational Modeling: Finite element analysis and biomechanical modeling of the abdominal wall can simulate the effects of weight loss, BTA, or PPP on fascial tension, guiding optimization strategy selection and predicting closure feasibility [143].

Augmented Reality Surgical Planning: Integration of 3D reconstructions with augmented reality platforms allows surgeons to visualize hernia anatomy, plan mesh positioning, and optimize operative approach preoperatively [144].

### 8.4. Biomarker-Guided Optimization

Emerging research suggests that biomarkers may guide personalized optimization. Specific metabolomic signatures associated with wound healing capacity and hernia recurrence risk may allow identification of patients requiring intensive preoperative optimization [145].

Identification of specific inflammatory markers might indicate the proper timing for hernia repair: Serial monitoring of CRP, IL-6, and other inflammatory biomarkers during GLP-1 therapy may help determine optimal surgical timing when inflammatory burden is minimized [79].

Non-invasive assessment of fascial quality through elastography or other imaging modalities may help determine readiness for repair and predict recurrence risk [146].

### 8.5. Microbiome and Preoperative Optimization

Emerging evidence suggests that the gut microbiome influences both metabolic health and surgical outcomes. Recent studies demonstrate that GLP-1 receptor agonists alter gut microbiome composition, potentially contributing to metabolic improvements and anti-inflammatory effects [147].

Specific microbiome signatures may predict surgical site infection risk, wound healing capacity, and response to metabolic optimization interventions. Targeted prebiotic or probiotic interventions combined with metabolic optimization may synergistically improve perioperative outcomes—an area requiring further investigation [148,149].

## 9. Future Research Directions and Unanswered Questions

Although preliminary evidence suggests potential benefits of GLP-1 and GLP-1/GIP agonists in preoperative optimization, several important knowledge gaps remain. To date, no hernia-specific randomized controlled trials have defined optimal dosing, treatment duration, or discontinuation protocols. Data on patient-reported outcomes, quality of life, and long-term recurrence rates following GLP-1–facilitated optimization are also lacking.

Comparative effectiveness between pharmacologic and mechanical strategies has not been established, and the ideal sequencing or combination of these interventions remains empirical. Standardized patient selection criteria and dedicated cost-effectiveness analyses are urgently needed to guide individualized optimization approaches.

In parallel, the integration of Biomechanically Controlled Repair (BCR) introduces an additional dimension to future research. Although early outcomes demonstrate markedly reduced recurrence rates and improved long-term durability, robust mechanistic data explaining how individualized force-distribution modeling interacts with preoperative metabolic and mechanical interventions are lacking. Key unanswered questions include whether prehabilitation strategies modify the biomechanical parameters used in BCR planning, how mesh vector optimization performs across heterogeneous tissue qualities, and whether BCR should be selectively combined with pharmacologic or mechanical optimization to achieve maximal benefit.

Mechanistic understanding is likewise incomplete. The relative contributions of systemic weight loss, anti-inflammatory activity, and local tissue remodeling to improved healing are unclear, and the absence of biomarkers limits the ability to predict treatment response.

Emerging interest in preoperative collagen optimization adds another layer of complexity to the evolving landscape. Despite biological plausibility and increasing use of targeted micronutrient supplementation, collagen peptides, and anabolic support strategies, no controlled studies have defined optimal dosing regimens, treatment duration, or the patient populations most likely to benefit. Furthermore, the relationship between systemic collagen biomarkers and hernia-specific healing responses remains largely unexplored, representing a key frontier for biomarker-guided personalization of prehabilitation.

Evidence on long-term outcomes—such as the durability of weight loss, recurrence beyond five years, and functional recovery after surgery—remains limited. Future studies should prioritize prospective, adequately powered trials that integrate clinical, mechanistic, and patient-centered endpoints to define the true value of GLP-1–based preoperative optimization.

Future investigations must also address the economic and practical barriers associated with implementing optimization strategies for patients requiring abdominal wall reconstruction. Key uncertainties remain regarding the overall cost burden of prolonged pharmacologic or procedural prehabilitation, the extent to which insurance coverage and healthcare system resources influence equitable access, and how financial constraints may differentially affect high-risk populations. Moreover, little is known about long-term adherence to therapies such as GLP-1 receptor agonists in the preoperative setting, particularly when treatment extends over several months and may be discontinued once surgical symptoms improve. Understanding how cost, accessibility, and adherence intersect will be essential for developing sustainable, patient-centered models of care that can be broadly implemented across diverse clinical environments.

Given the increasing array of available optimization strategies, comparative effectiveness research is urgently needed. Head-to-head trials should evaluate GLP-1 therapy against BTA and PPP in multimodal candidates, assess the benefits of combined approaches versus monotherapy, and compare outcomes of BCR performed after optimization with BCR alone and with conventional repair techniques. Determining whether sequential or concurrent optimization yields superior outcomes remains a high priority.

Finally, despite expanding technical and clinical research, patient-reported outcomes and quality-of-life measures remain underrepresented in the current literature. Future studies must incorporate assessments of quality of life during extended optimization periods, patient preferences regarding pharmacologic versus procedural interventions, functional recovery trajectories, and the financial burden associated with prolonged preoperative treatment. Integrating these perspectives will be essential for aligning optimization strategies with patient-centered care and for ensuring equitable access as the field continues to evolve.

## 10. Conclusions

The landscape of preoperative optimization for abdominal wall hernia repair is evolving rapidly, driven by the convergence of three forces: the global epidemics of obesity and diabetes, technological advances in pharmacotherapy and procedural techniques, and growing recognition that traditional surgical approaches yield suboptimal outcomes in high-risk patients.

GLP-1 receptor agonists and dual GLP-1/GIP agonists represent a transformative addition to the preoperative management options. Their ability to produce substantial weight loss (15–20%), improve glycemic control (HbA1c reductions of 1.5–2.5%), reduce systemic inflammation, and provide cardiovascular protection creates a compelling metabolic foundation for successful hernia repair. Emerging evidence from surgical cohorts suggests meaningful reductions in wound complications, infections, thromboembolic events, and readmissions, though hernia-specific randomized trials remain absent.

Botulinum toxin A injections and progressive pneumoperitoneum maintain critical roles in the mechanical preparation of complex loss-of-domain hernias. By directly addressing abdominal wall compliance and domain deficits, these techniques enable successful repair of hernias that would otherwise be prohibitively complex or dangerous. The synergistic combination of BTA and PPP represents the current standard for mechanical optimization in giant hernias.

The emerging paradigm embraces personalized, multimodal optimization strategies tailored to individual patient phenotypes and hernia characteristics. Rather than viewing pharmacologic and mechanical approaches as competing alternatives, the field is moving toward integrated protocols that combine systemic metabolic conditioning with targeted mechanical preparation when both are indicated.

Alongside these systemic and mechanical strategies, BCR is reshaping operative decision-making by providing a quantitative framework for mesh selection, overlap, and vector orientation based on patient-specific force mapping. By minimizing strain at the repair site and standardizing the biomechanical quality of the reconstruction, BCR has demonstrated substantial reductions in recurrence and chronic pain. Its integration into multimodal optimization pathways may ultimately bridge the gap between preoperative conditioning and durable surgical outcomes.

The evidence base, while still incomplete, provides sufficient signal to justify clinical adoption of optimization strategies in appropriately selected patients. Surgeons and healthcare systems should begin implementing these approaches within frameworks that enable careful outcome tracking and continuous quality improvement. Simultaneously, the research community must prioritize rigorous evaluation through well-designed trials to definitively establish efficacy, optimize protocols, and guide evidence-based practice.

An additional emerging pillar of comprehensive optimization is the targeted enhancement of collagen quality and extracellular matrix integrity. Nutritional and molecular interventions aimed at improving collagen synthesis, cross-linking, and tissue remodeling may complement both BCR and metabolic optimization by strengthening the biological substrate on which repairs depend. Although clinical evidence remains preliminary, the concept of preparing the abdominal wall at a molecular level aligns closely with the broader shift toward personalized and biology-informed surgical care.

Ultimately, the goal transcends technical success in closing fascial defects. By bridging metabolism, mechanics, and outcomes, comprehensive preoperative optimization offers the promise of not only better hernia repairs, but healthier patients—transforming a single surgical episode into an opportunity for lasting metabolic improvement and enhanced quality of life.

## Figures and Tables

**Table 1:** Comparison between preoperative optimization methods.

Feature	Pharmacologic Optimization (GLP-1/GIP Agonists)	Mechanical Optimization (BTA/PPP)	Preoperative Collagen Optimization	Biomechanically Controlled Repair (BCR)
**Primary target**	Systemic metabolic dysfunction	Local abdominal wall mechanics	Extracellular matrix quality and collagen synthesis	Force distribution, tissue load-sharing, and mesh vector optimization
**Mechanisms**	Weight loss, improved glycemic control, inflammation reduction, cardiovascular protection	Muscle elongation (BTA), intra-abdominal cavity expansion and compliance enhancement (PPP)	Nutrient supplementation (collagen peptides, vitamin C, arginine, zinc), reduced collagen degradation, improved ECM turnover	Preoperative CT-based biomechanical analysis, personalized mesh shape/overlap, optimized suture vectors
**Physiologic effects/Benefits**	↓ Intra-abdominal pressure, improved tissue healing, ↓ infection risk, cardiovascular protection	Facilitated fascial closure, reduced compartment syndrome risk	↑ Collagen type I production, improved tensile strength, enhanced wound healing, better mesh incorporation	↓ Mechanical strain at repair site, ↓ recurrence rate, improved long-term durability, ↓ chronic pain
**Timeline to effect**	Months (typically 3–12 months for optimal response)	Weeks (≈2–4 weeks for BTA; 1–3 weeks for PPP)	Weeks to months (2–8 weeks depending on regimen)	Immediate intraoperatively; long-term durability over months–years
**Ideal candidates**	Obese or diabetic patients (BMI > 35 kg/m^2^ or HbA1c > 7%) with any hernia type	Loss-of-domain hernias, large fascial defects > 10 cm, VIH/VAC > 20%, irrespective of metabolic status	Patients with suspected collagen dysregulation, recurrent hernias, smokers, elderly, poor nutritional status	All complex hernias, recurrent hernias, large defects, patients with high mechanical strain risk
**Representative goal**	Metabolic and systemic optimization prior to surgery	Anatomic and biomechanical optimization enabling tension-free closure	Improve ECM integrity and healing capacity before and after repair	Achieve physiologically balanced force distribution to minimize recurrence
**Complementarity**	Addresses systemic contributors to poor healing and recurrence	Addresses local mechanical tension; synergistic when combined with pharmacologic optimization	Enhances biological capacity to maintain repair; synergistic with BCR and metabolic optimization	Integrates mechanical and physiologic data; synergistic with PPP/BTA and metabolic optimization

## Data Availability

No new data were created or analyzed in this study.
